# Association between smoking and hypertension under different PM_2.5_ and green space exposure: A nationwide cross-sectional study

**DOI:** 10.3389/fpubh.2022.1026648

**Published:** 2022-11-17

**Authors:** Qihao Chen, Xuxi Ma, Yan Geng, Jingling Liao, Lu Ma

**Affiliations:** ^1^Department of Biostatistics, School of Public Health, Wuhan University, Wuhan, China; ^2^Department of Global Health, School of Public Health, Wuhan University, Wuhan, China; ^3^Department of Nutrition and Food Hygiene, School of Public Health, Medical College, Wuhan University of Science and Technology, Wuhan, China

**Keywords:** smoking, hypertension, PM_2.5_, green space, air pollution

## Abstract

**Background:**

Smoking has been widely reported to have a significant relationship with hypertension, but the past description of this relationship has not been uniform. In addition, there has been a lack of research to discuss the impact of environmental exposure on the relationship between smoking and hypertension. Therefore, this study estimates the association between smoking and hypertension in middle aged and elderly people in China under different PM_2.5_ (fine particulate matter) concentrations and the green space exposure conditions.

**Methods:**

Individual sample data from the China Health and Retirement Longitudinal Study in 2018 and the long-term average exposure concentration of fine particles and green space exposure for all participants were used with a multilevel binary logistic mixed effects model. Adjustments were made for sociodemographic characteristics and other health behaviors including drinking, physical activity, and social activity. The normalized difference vegetation index (NDVI) and PM_2.5_ concentration stratification were assigned with the median of the population exposure concentration as the dividing line, and the dual environmental factor stratification was assigned in combination with the two types of environmental exposure. The analysis was also stratified using age groups.

**Results:**

A total of 10,600 participants over the age of 45 were included in the study. The effects of smoking on hypertension were diverse under different environmental exposure conditions. There was a significant relationship between smoking behavior and hypertension in the Low-NDVI group, and the effect value of this relationship was significantly different from that in the High-NDVI group. Furthermore, for respondents exposed to low green spaces and high PM_2.5_ environments at the same time (Low-NDVI/High-PM_2.5_ group), their smoking behavior may lead to an increase in the risk of hypertension. In addition, the risk of hypertension caused by smoking in the middle-aged (45–64) was significant under low green space exposure, but the effect difference between the different age groups was not significant.

**Conclusions:**

The relationship between smoking and hypertension was different under different environmental exposure conditions. Exposure to low green spaces may strengthen the association between smoking and hypertension risk. When participants were exposed to both low green spaces and high PM_2.5_ concentrations, the risk of hypertension caused by smoking was significantly higher than that of those who were exposed to high green spaces and low PM_2.5_ concentrations.

## Introduction

Smoking has been widely reported as a risk factor for cardiovascular disease (CVD) (1, 2), while smoking cessation and tobacco consumption control are regarded as important means to prevent CVDs such as hypertension and coronary heart disease (3). Published studies support that atherosclerosis caused by smoking may increase the risk of hypertension (4), and nicotine may lead to a rise in blood pressure (BP) through a variety of biological mechanisms such as symptomatic action and modulation of the renin angiotensin system (5).

Although a significant association between smoking and hypertension is widely recognized, the association strength and the effect description among smokers in previous studies have been inconsistent. For this reason, previous studies have tried to control the confounding factors in the association analysis between smoking and hypertension using the body mass index (BMI), demography, smoking behavior details, and other aspects, but the results obtained were still different. A study on men reported that the relative risk of hypertension of former smokers and current smokers reached 1.08 and 1.15, respectively (6). However, a cross-sectional study conducted only in men also found that there was a more significant relationship between former smokers and hypertension, with an odds ratio (OR) of 1.48 (95 CI%: 1.01, 2.18), while the risk of hypertension in current smokers was not significant (7). A longitudinal population-based study supported that the abdominal obesity population had a higher risk of hypertension due to smoking. The OR of former smokers compared with current smokers was 1.62 (95, CI%: 1.08, 1.41) (8). However, a cross-sectional study in France reported that there was a significant relationship between current smokers and hypertension, and claimed that the BMI was independent of this relationship (9). Zhang et al. also believe that the potential confounding effect from body weight cannot totally explain the inconsistent findings (10). A study conducted in Iran suggested that a significant socioeconomic status (SES)-smoking association may determine increasing blood pressure (11), while another study that examined education, the living area, and other SES factors as covariate controls found that smoking had no significant relationship with hypertension (12).

As an important risk factor related to hypertension, environmental factors, such as air pollution and green space, have been widely associated with hypertension in the field of environmental epidemiology. However, most previous studies on smoking and hypertension lacked the control of environmental exposure. As an important pollutant in the atmosphere, PM_2.5_ has frequently appeared in hypertension risk factor studies (13, 14), and multiple studies have focused on the elderly, supporting the possibility that smoking might lead to an increase in BP (15, 16). In addition, studies regarding long-term exposure to green space have indicated that the odds of hypertension are related to the green space exposure (17), and some studies have claimed that living near green spaces may reduce the risk of hypertension (18, 19). Under the premise that environmental factors have potential effects on hypertension, the lack of control over environmental factors in the analysis may have led to biased effect descriptions.

Therefore, to further explain the relationship between smoking and hypertension, it is necessary to explore the risk difference of smoking on hypertension under complex environmental exposure. The China Health and Retirement Longitudinal Study (CHARLS) used a multilevel binary logistic mixed effects regression to analyze the risk of hypertension caused by smoking under different PM_2.5_ concentrations and green space exposure, attempting to provide a new perspective for the study of hypertension risk factors. In addition, a nationwide study reported that the tobacco dependence of middle-aged people in China was significantly higher than that of the elderly (20). Hence, an age stratified analysis under different environmental conditions is also included in our study.

## Materials and methods

### Study population

The information of study population was collected from the China Health and Retirement Longitudinal Study (CHARLS). CHARLS aims to collect a set of high-quality micro-data representing families and individuals aged 45 and over in China that provide data support for an analysis of aging of the Chinese population and promote the interdisciplinary research of aging (21). CHARLS covers 150 county-level units (the latest survey resulted in 2018 covering 125 cities), 450 village level units, and nearly 20,000 people in approximately 10,000 households. The CHARLS national baseline survey was conducted in 2011, and three follow-up investigations were repeated in 2013, 2015, and 2018. The datasets are available for researchers after registration and can be obtained on the corresponding website.

It should be noted that the propose of this study is to assess the difference in the impact of smoking on hypertension under different long-term environmental conditions, while according to the Air Pollution Prevention and Control Action Plan proposed by the State Council of China, China included all of the prefecture level cities in the PM_2.5_ measurement on January 1, 2015 (22). Therefore, to obtain complete long-term exposure concentration data of PM_2.5_, CHARLS 2018 conducted from July 2018 to September 2018 was chosen as the data support of this cross-sectional study.

### Health data

Individual information on the smoking status, hypertension diagnosis, other health behaviors and demographic data were collected from the China Health and Retirement Longitudinal Study—Baseline Questionnaire, which was one of a series of questionnaires in CHARLS.

#### Smoking status

For smoking status, respondents were described as “never smoking”, “current smoker”, and “former smoker”. First, for question DA059 (“Have you ever chewed tobacco, smoked a pipe, smoked self-rolled cigarettes, or smoked cigarettes/cigars?”), respondents were divided into never smoking and people with smoking history. Second, according to DA061 (“Do you still have the habit, or have you totally quit?”), people with smoking histories were further divided into “current smoker” and “former smoker”, which means that smoking status was described as a categorical variable in this study.

#### Hypertension diagnosis

The diagnosis basis of hypertension was obtained from DA007 [“Have you been diagnosed with (one of the following chronic diseases) by a doctor?”] that included hypertension, dyslipidemia, diabetes or high blood sugar, cancer or malignant tumor, chronic lung diseases, liver disease, heart disease, stroke, kidney disease, stomach or other digestive disease, emotional, nervous, or psychiatric problems, memory-related disease, arthritis or rheumatism, or asthma. In this study, hypertension was defined as individuals who reported having been diagnosed with hypertension.

#### Covariates

The demographic information included age, sex, and education level (BD001: “What is the highest level of education you have attained?” described as under primary school, primary school, middle school or above), and the per capita gross domestic products (GDPs) of all cities in 2018 was collected from the statistical yearbook of each province (23) were considered as covariates to be controlled. As health behavior variables mentioned in previous hypertension relationship studies (21, 24, 25), alcohol drinking (DA067: “Did you drink any alcoholic beverages, such as beer, wine, or liquor in the past year? How often?”, described as drink more than once a month, drink but less than once a month, never drink), sleep time (DA049: “during the past month, how many hours of actual sleep did you get at night?”), physical activity (DA051: “during a usual week, did you do any physical activity for at least 10 minutes continuously?”, yes or no), and social activity (DA056: “have you done any of these activities below in the last month?”, yes or no) were included in the analysis. Daily cigarette consumption (DA063: “In one day about how many cigarettes do/did you consume?”) as a detailed variable of smoking behavior was also included in the analysis.

**Figure 1:**
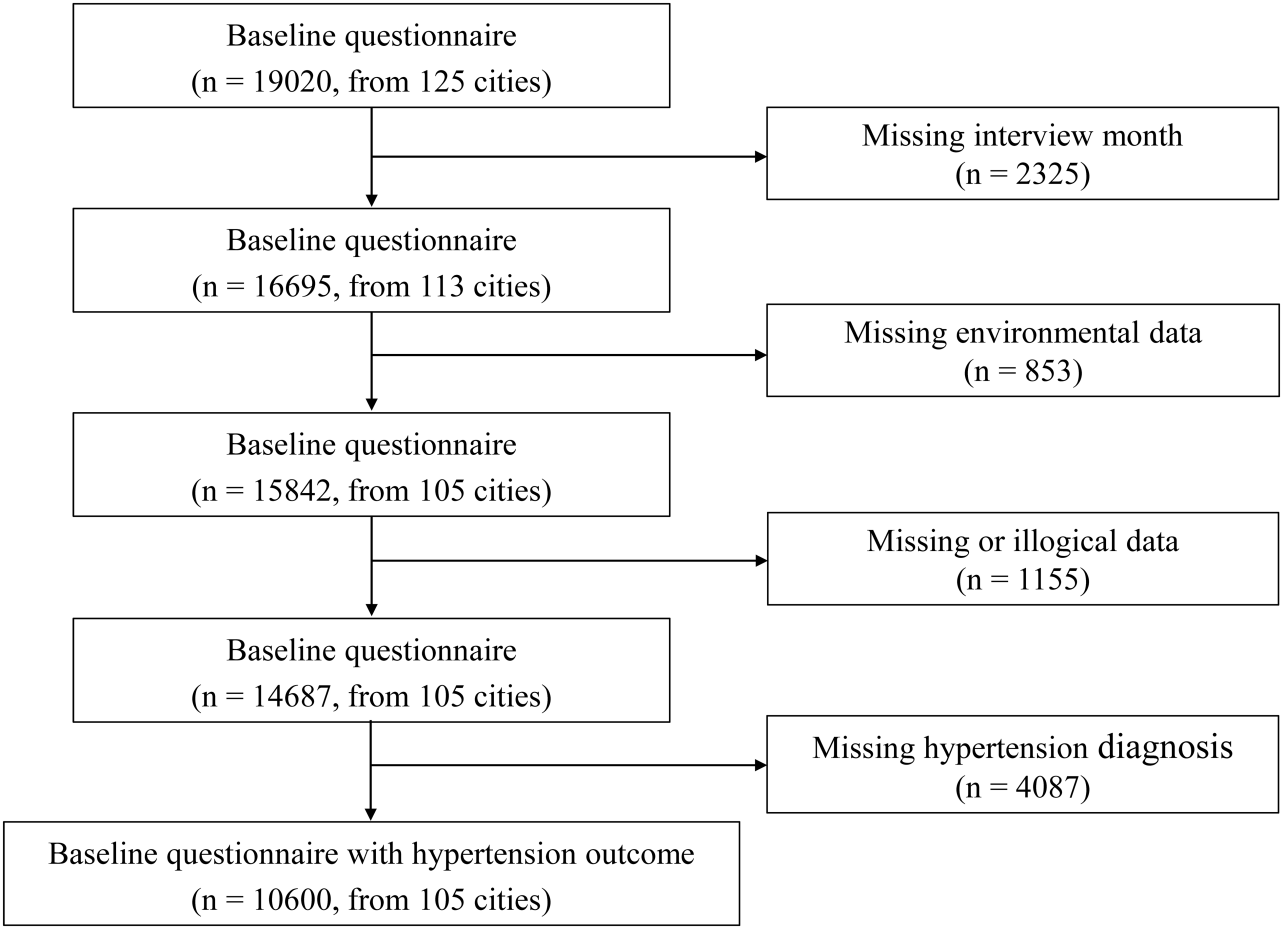
Flow chart of selection procedure.

We used the CHARLS2018 as the data support that included 19,020 respondents from 125 cities. Due to the need to define the one-year exposure period by the month of interview, 12 cities (Fuzhou City, Putian City, Ningde City, Zhangzhou City, Chengdu City, Guangan City, Kunming City, Baoshan City, Zhaotong City, Lijiang City, Lincang City, and Haidong City) that lacked the records of the month of interviews were not included in the analysis, and a total of 2,325 respondents were excluded. Of the remaining 113 cities, eight were not within the scope of the National Ambient Air Quality Monitoring Network. Hence, 853 respondents in these cities were excluded (Chaohu City, Chuxiong Yi Minority Autonomous Prefecture, Ganzi Tibetan Autonomous Prefecture, Liangshan Yi Autonomous Prefecture, Qiandongnan Miao and Dong Autonomous Prefectures, Qiannan Buyei and Miao Autonomous Prefectures, Xiangfan City, and the Xing ‘an League). We also excluded 1,155 respondents with missing or illogical data, such as respondents under 45 because the CHARLS does not systematically sample the population below age 45 (26). Finally, 4,087 respondents were excluded due to lack of hypertension diagnostic information, and our final sample included 10,600 respondents from 105 cities. According to the Chi square test, there was no significant difference in the prevalence of hypertension between the excluded 8,420 respondents and the final 10,600 respondents included in the study (*P* = 0.425). Figure 1 shows the specific screening process of the respondents in this study.

### Exposure assessment

#### Air pollution

The PM_2.5_ data were collected from the National Ambient Air Quality Monitoring Network established by the China National Environmental Monitoring Center. The monitoring network includes a total of 1,436 stations covering 338 cities in China that release daily average concentration data on PM_2.5_ and other air pollutants in each city in hourly terms. The mean daily average of all the fixed monitoring points in each city was considered as the daily PM_2.5_ concentration of the respondents. To explore the long-term exposure effect, we defined the one-year exposure period for each respondent using the last day of the interview month as the end point.

Based on obtaining the daily average concentration, we calculated the annual average concentration to describe the long-term exposure to PM_2.5_. In addition, we introduced O_3_ (ozone) and NO_2_ (nitrogen dioxide) as covariates in the model control because they have been reported as influencing factors of hypertension (27).

#### Green space

As a measure of the relative plant health in terrestrial habitats and landscapes, the normalized difference vegetation index (NDVI) is widely used as an index to evaluate the exposure level of green space (28). The NDVI quantifies the density of green vegetation based on the difference between the surface reflectance of near-infrared (NIR) and visible red (VISR) measured by satellite (29).


$$\begin{array}{l} NDVI=\frac{\left(NIR-VISR\right)}{NIR+VISR}. \end{array}$$

NDVI values vary between −1 and 1 where pixels with dense vegetation typically yield high positive numbers, while a negative NDVI value indicates that the ground is covered with clouds, water, or snow that are highly reflective of visible light. An NDVI equal to 0 means that the ground is rock or bare soil, and the NIR and VISR are approximately equal (30). Since this study has not discussed the environmental factors of water, the value of an NDVI <0 is null by default.

The NDVI data of this study were collected from the Moderate Resolution Imaging Spectroradiometer (MODIS). As the primary instrument that monitors the earth's surface onboard the earth observation systems Terra and Aqua (31), MODIS records data to analyze several environmental variables including greenness, and it has been widely used for global research on green spaces and other environmental factors (32). After data screening to determine the cities involved in the study, green space satellite images with spatial resolutions of 1 km ^*^ 1 km and time resolutions of one every 30 days (12 images per year) were used to extract the monthly average NDVI of all cities in study. With reference to the long-term exposure assessment of PM_2.5_, the average annual NDVI exposure concentration of each respondent was calculated with the month of the interview as the end point.

### Statistical analysis

#### Multilevel model

Respondent data from CHARLS contains two levels of variables: health and demographic factors at the individual level and environmental exposure factors and socio-economic factors at the regional level. This means that the data in this study followed a hierarchical structure (25). Considering that the traditional binary logistic regression model only describes effects from a single level, multilevel binary logistic mixed effects models were used to examine the relationship between smoking and hypertension under different environmental conditions (33). For all analysis, we constructed an unadjusted model (Model 0), a model adjusted for age, sex, education level, the per capita GDP, alcohol drinking, daily cigarette consumption, sleep time, physical activity, and social activity (Model 1), and a model additionally adjusted for environmental factors included the NDVI, PM_2.5_, O_3_, and NO_2_ (Model 2). Generalized variance inflation factors (GVIFs) were used to quantify the multicollinearity between the environmental exposures (30).

#### Stratified analysis

Based on the significant relationship between smoking and hypertension observed in the main model, a stratified analysis of the environmental factors was conducted. By using the median annual average exposure concentration of the NDVI and PM_2.5_ of each respondent as the dividing line, a single environmental factor stratified analysis for two environmental factors was conducted separately. To verify the comparability of the odds ratios under different regressions, we conducted a significant difference test on the regression coefficients of the different concentration groups. Douglas G (34) mentioned that the standard deviation of the difference between the two estimates has the relationship of the flow equation that follows:


$$\begin{array}{l} SE\left(d\right)=\sqrt{SE{\left(E_{1}\right)}^{2}+SE{\left(E_{2}\right)}^{2}}, \end{array}$$

where ***E***_**1**_ and ***E***_**2**_ represent the estimated value of smoking for the increased risk of hypertension in the different groups of environmental factor exposure concentrations. After obtaining the standard deviation of the estimated value difference, ***SE(d)***, the test of interaction was based on the flow equation:


$$\begin{array}{l} Z=\frac{d}{SE\left(d\right)}. \end{array}$$

The ratio ***Z*** gives a test of the null hypothesis that in the population the difference d is zero by comparing the Z score to the standard normal distribution. For this study, the *P*-value obtained by referring the Z score to the normal distribution table was used to described whether the difference between the different pollution concentration groups was significant. In addition, this study also conducted a stratified analysis of age under different single environmental factors. Single environmental factor stratified analyses were conducted in the different age groups (45–64/>64) to observe the difference of their effects. Tests for the difference in the regression estimates in the environmental concentration stratification and age stratification were conducted.

Furthermore, we combined the grouping conditions of the single environmental factor stratified analysis and conducted a dual environmental factor stratified analysis. The double environmental factor stratification describes the level of the NDVI and PM_2.5_ exposure of respondents for the entire population during the study period. All respondents were divided into four groups: Low NDVI / Low PM_2.5_ group, Low NDVI / High PM_2.5_ group, High NDVI / Low PM_2.5_ group, and High NDVI / High PM_2.5_ group. For example, if a respondent was in the Low NDVI / Low PM_2.5_ group, this meant that both the respondent 's exposure to PM_2.5_ and green space during the one-year exposure period ending at the interview date were lower than the median annual exposure concentration of the study population. For all groups that observed a significant association between smoking and hypertension, the difference between their estimates and each other group were tested using the Z score and reported in the form of the *P-*value (P for ORs).

#### Sensitivity analysis

To evaluate the robustness of our results, we conducted a sensitivity analysis by defining two different exposure periods (a half-year and 2 years). A single environmental factor stratification analysis and dual environmental factor stratification analysis were conducted during two different exposure periods. All of the statistical analyses were conducted using R version 4.2.1, and two-sided *p*-values (*p* < 0.05) were considered statistically significant.

## Results

### Descriptive statistics

The basic characteristics of the study participants are summarized in Table 1. This study included 10,600 middle-aged or older respondents with a mean age of 59.82 years and an approximately equal sex distribution (46.82% males and 53.18% females). A total of 61.12% of the respondents had never smoked, while 26.85% were current smokers, and former smokers accounted for 12.03% of all the respondents. There were 1,584 participants with hypertension, and this indicated that the prevalence of the study population was 14.94%. The daily cigarette consumption of the survey population was 6.78, and the average GDP per capita reached 52,852 yuan. For the NDVI concentration stratification, significant differences were observed for education level and social activity. While for the PM_2.5_ concentration stratification, education level, smoking status, drinking status, physical activity, and GDP per capita showed significant distribution differences. The distribution of daily cigarette consumption and the prevalence of hypertension did not show significant differences in both the NDVI and PM_2.5_ concentration stratification (*P* > 0.05).

**Table 1:** Descriptive statistics of the study population.

**Variable**	**Total (*n =* 10,600)**	**Low NDVI (*n =* 5,258)**	**High NDVI (*n =* 5,342)**	*P-*value^*^	**Low PM_2.5_ (*n =* 5,248)**	**High PM_2.5_ (*n =* 5,352)**	*P*-value^*^
Prevalence rate	1,584 (14.94%)	780 (14.83%)	804 (15.05%)	0.776	759 (14.46%)	825 (15.41%)	0.178
Age (mean ± SD)	59.82 ± 9.79	59.85 ± 9.79	59.79 ± 9.80	0.151	59.96 ± 9.93	59.68 ± 9.66	0.750
Gender				0.299			0.190
Male	4,963 (46.82%)	2,489 (47.34%)	2,474 (46.31%)		2,423 (46.17%)	2,540 (47.46%)	
Female	5,637 (53.18%)	2,769 (52.66%)	2,868 (53.69%)		2,825 (53.83%)	2,812 (52.54%)	
Education level				0.000			0.000
Under primary school	4,021 (37.93%)	1,829 (34.79%)	2,192 (41.03%)		1,931 (36.79%)	2,090 (39.05%)	
Primary school	2,397 (22.61%)	1,225 (23.3%)	1,172 (21.94%)		1,256 (23.93%)	1,141 (21.32%)	
Middle school or above	4,182 (39.45%)	2,204 (41.92%)	1,978 (37.03%)		2,061 (39.27%)	2,121 (39.63%)	
Smoking status				0.823			0.017
Never Smoking	6,479 (61.12%)	3,228 (61.39%)	3,251 (60.86%)		3,219 (61.34%)	3,260 (60.91%)	
Former smoker	1,275 (12.03%)	632 (12.02%)	643 (12.04%)		586 (11.17%)	689 (12.87%)	
Current smoker	2,846 (26.85%)	1,398 (26.59%)	1,448 (27.11%)		1,443 (27.50%)	1,403 (26.21%)	
Daily cigarette consumption	6.78 ± 11.75	6.935 ± 9.795	6.619 ± 9.795	0.284	6.697 ± 9.932	6.853 ± 9.657	0.698
Drinking status				0.448			0.005
Never drink	6,863 (64.75%)	3,383 (64.34%)	3,480 (65.14%)		3,385 (64.50%)	3,478 (64.99%)	
Light drinking	2,862 (27.00%)	1,424 (27.08%)	1,438 (26.92%)		1,385 (26.39%)	1,477 (27.60%)	
Habitual drinking	875 (8.25%)	451 (8.58%)	424 (7.94%)		478 (9.11%)	397 (7.42%)	
Sleep time (mean ± SD)	6.27 ± 1.89	6.30 ± 1.87	6.24 ± 1.92	0.586	6.28 ± 1.90	6.26 ± 1.89	0.106
Physical activity				0.905			0.011
No	949 (8.95%)	473 (9.00%)	476 (8.91%)		432 (8.23%)	517 (9.66%)	
Yes	9,651 (91.05%)	4,785 (91.00%)	4,866 (91.09%)		4,816 (91.77%)	4,835 (90.34%)	
Social activity				0.000			0.889
No	4,676 (44.11%)	2,150 (40.89%)	2,526 (47.29%)		2,311 (44.04%)	2,365 (44.19%)	
Yes	5,924 (55.89%)	3,108 (59.11%)	2,816 (52.71%)		2,937 (55.96%)	2,987 (55.81%)	
Per capita GDP (yuan, mean ± SD)	55,852.00 ± 0.43	58,130.40 ± 0.52	53,609.42 ± 0.46	0.829	55,915.61 ± 0.55	55,789.62 ± 0.43	0.000

* The P-value between different exposure concentration groups was calculated using the Chi square test for the categorical variables and the two-sample unpaired t test for the continuous variables.

**Figure 2:**
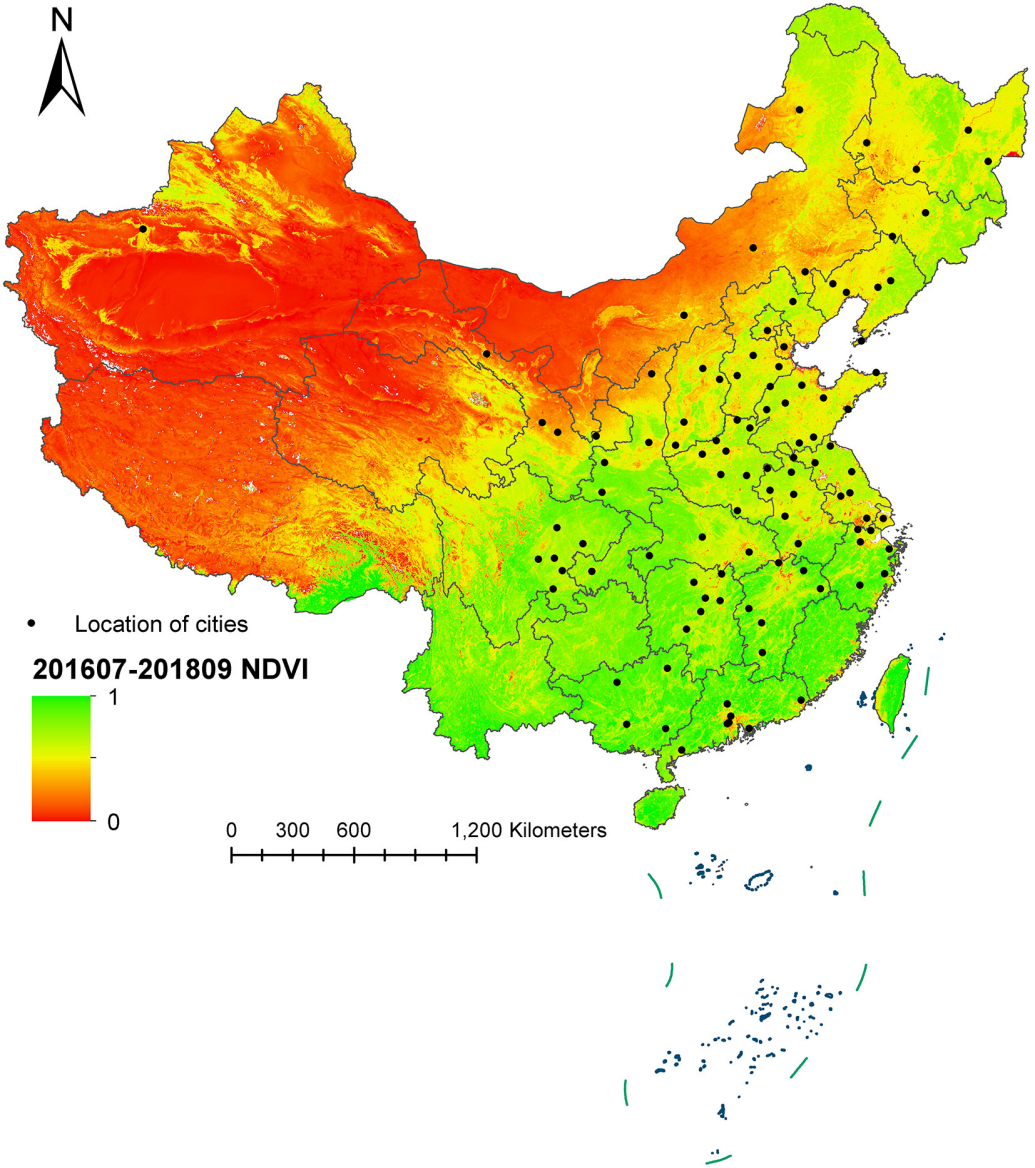
Average concentration distribution of the NDVI during July 2016 to September 2018 in China.

The summary statistics on the environmental data during July 2016 to September 2018 of all cities included in this study are shown in Table 2. The average PM_2.5_ concentration of 105 cities from July 2016 to September 2018 was 49.09 μg/m^3^, with a median concentration of 44.86 μg/m^3^. Shijiazhuang City had the highest average PM_2.5_ concentration, reaching 90.70 μg/m^3^, while Hulunbeier City showed the lowest long-term average exposure concentration of PM_2.5_. For O_3_ and NO_2_, the average concentrations reached 95.98 and 29.06 μg/m^3^, respectively, during the study period. The average value of the NDVI reached 0.54, with a median of 0.53. Anyang City and Siping City were the cities with the lowest and highest NDVI exposures among all of the cities, with NDVI values of 0.13 and 0.73, respectively. Figure 2 shows the average concentration distribution of the NDVI in China during study period. It was found that the NDVI of the central and southern region sin China were higher than that of other regions, and most of the cities included in the study were located in these two areas. Some cities were located in the northeast region, and the NDVIs of this region were lower than that of the south.

**Table 2:** Summary statistics of the air pollutants and NDVI during the study period.

**Variable**	**Mean ±SD**	**Percentiles**					**IQR**
		**Min**	**25th**	**50th**	**75th**	**Max**	
PM_2.5_	49.09 ± 13.86	20.18	38.47	44.86	58.50	90.70	20.02
NO_2_	33.86 ± 8.70	17.38	26.82	34.18	39.94	54.12	13.12
O_3_	97.11 ± 10.17	70.16	90.50	98.48	104.62	114.99	14.12
NDVI	0.54 ± 0.11	0.13	0.44	0.50	0.60	0.73	0.16

### Primary model results

Table 3 shows the primary model results of the relationship between hypertension and smoking. In the unadjusted Model 0, the smoking behavior of the current smokers and former smokers showed a significant relationship with hypertension, and the OR reached 1.064 (95 CI%: 1.012, 1.118) and 1.082 (95 CI%: 1.021, 1.146), respectively. After controlling for demographic factors, such as gender and age, and the health behavior covariates, such as physical activity and social activity, the relationship between the current smokers and hypertension in Model 1 showed a marginal significance (*P* = 0.096), and the OR reached 1.022 (95 CI%: 0.996, 1.048). The smoking behavior of former smokers was still statistically significant for the risk of hypertension (*P* = 0.011), and the risk of hypertension increased by 3.87% (95 CI%: 1.009, 1.070) compared with those who had never smoked. In Model 2 that controls for air pollutants and the NDVI, the correlation strength and significance between smoking behavior and hypertension of former smokers and current smokers increased slightly. The risk of hypertension in former smokers increased by 3.92% (95 CI%: 1.009,1.070) compared with those who had never smoked, and the relationship between former smokers and hypertension was significantly enhanced after controlling for environmental factors (*P* = 0.009). For current smokers, the OR reached 1.024 (95 CI%: 0.998, 1.051) in Model 2, and the relationship between current smokers and hypertension was still marginal significant (*P* = 0.071).

**Table 3:** Odds ratios (OR) of hypertension (with 95% confidence intervals, 95%CI) associated with smoking.

**Model**	**Covariates**	**Smoking status**	**OR (95%CI)**
Crude	Unadjusted	Never smoking	-
		Former smoker	1.082 (1.021,1.146)^***^
		Current smoker	1.064 (1.012,1.118)^**^
Basic	Age, sex, education level, the per capita GDP ^a^, alcohol drinking, daily cigarette consumption, sleep time, physical activity and social activity	Never smoking	-
		Former smoker	1.039 (1.009,1.070)^**^
		Current smoker	1.022 (0.996,1.048)^*^
Main	Basic model + NDVI + PM_2.5_ + O_3_ + NO_2_	Never smoking	-
		Former smoker	1.039 (1.009,1.070)^***^
		Current smoker	1.024 (0.998,1.051)^*^

a Per capita GDP has been logarithmically converted.

(1)

*** p < 0.01;

** p < 0.05;

* p < 0.10 (2) In all models, the GVIFs were < 1.785.

### Single environmental factor stratified results

By using the median annual average exposure concentration of the NDVI and PM_2.5_ of the respondents as the dividing line, a single environmental factor stratified analysis was conducted, and the results are presented in Table 4. The smoking behavior of the current smokers and former smokers was significantly related to the risk of hypertension in the different NDVI exposure levels. In the Low-NDVI group, the relationship between smoking behavior and hypertension of the current smokers and former smokers was statistically significant (*P* < 0.05), with the ORs reaching 1.058 (95 CI%: 1.020, 1.097) and 1.071 (95 CI%: 1.026, 1.117), respectively. However, in the High-NDVI group, no significant association with hypertension was observed in both current smokers and former smokers. Table 4 also reports the significance of the OR difference between the current smokers and former smokers in the different NDVI concentration groups. The *P*-value of the OR difference of the current smokers was 0.011, which means that there was a significant difference in the risk of hypertension among current smokers in the different NDVI exposure environments. For former smokers, the OR difference of the different NDVI groups was marginally significant (*P* = 0.059). Multicollinearity of exposures was not an issue in the NDVI stratified analysis (the GVIF values were below 1.808).

**Table 4:** ORs of hypertension (with 95% confidence intervals, 95%CI) associated with smoking stratified by a single environmental factor.

**Variable**	**By NDVI** ^**a**^					**By PM**_**2.5**_ ^**b**^				
	**Low-NDVI (*****n** =* **5,258)**		**High-NDVI (*****n** =* **5,342)**		**P for ORs**	**Low-PM**_**2.5**_ **(*****n** =* **5,248)**		**High-PM**_**2.5**_ **(*****n** =* **5,352)**		**P for ORs**
	***n***	**OR (95%CI)**	***n***	**OR (95%CI)**		***n***	**OR (95%CI)**	***n***	**OR (95%CI)**	
Never smoking	3,228	-	3,251	-		3,219	-	3,260	-	
Former smoker	632	1.071 (1.026,1.117)^***^	643	1.012 (0.972,1.054)^*^	0.059	586	1.026 (0.985,1.069)	689	1.052 (1.008,1.097)^**^	0.406
Current smoker	1,398	1.058 (1.020,1.097)^***^	1,448	0.990 (0.955,1.027)^**^	0.011	1,443	1.017 (0.981,1.055)	1,403	1.029 (0.992,1.068)	0.645

a Gender, education level, alcohol consumption, daily cigarette consumption, social activity, physical activity, sleep time, per capita GDP, NO_2_, PM_2.5_, and O_3_ were controlled as covariates in the model.

b Gender, education level, alcohol consumption, daily cigarette consumption, social activity, physical activity, sleep time, per capita GDP, NDVI, NO_2_, and O_3_ were controlled as covariates in the model.

(1)

*** p < 0.01;

** p < 0.05;

* p < 0.10 (2) In all models, the GVIFs were < 1.808. (3) P for ORs < 0.05 means that there was a significant difference in the ORs of the different exposure concentration groups.

For the stratified analysis of PM_2.5_, we did not observe a significant effect of the current smokers and former smokers on the risk of hypertension in the Low-PM_2.5_ group. For the former smokers with long-term PM_2.5_ exposure above the median of the population, the risk of hypertension caused by smoking behavior was statistically significant, with an OR of 1.052 (95 CI%: 1.008, 1.097). Although the current smokers in the High-PM_2.5_ group showed a trend of an increased risk for hypertension, the association was not statistically significant. It should be emphasized that in the test of the OR difference, the OR difference of the hypertension risk among former smokers in the different PM_2.5_ groups was not significant (*P* = 0.406). After GVIF verification, all models of the PM_2.5_ stratified analysis did not have multicollinearity.

**Figure 3:**
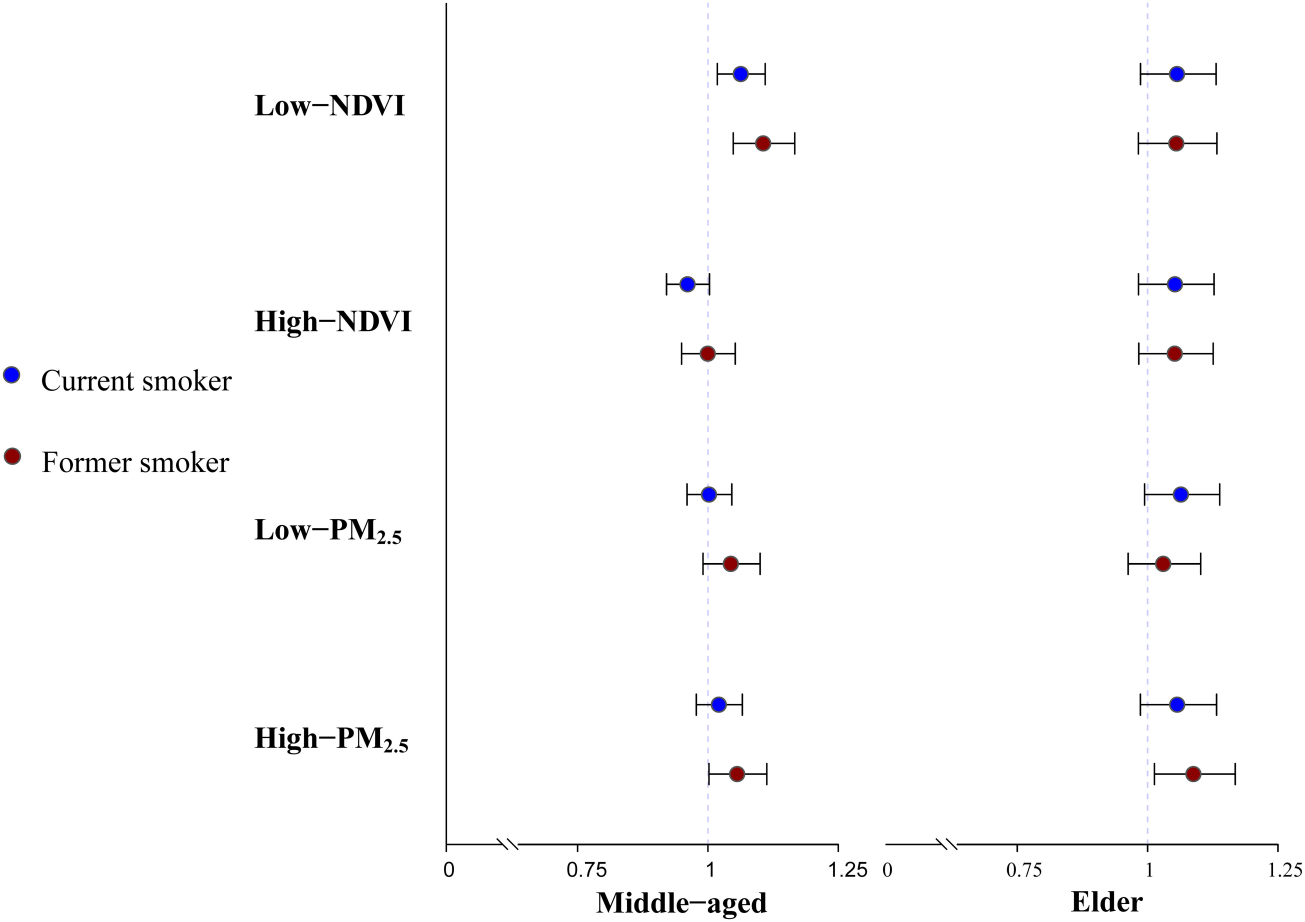
Single environmental factor analysis among mid-age and elder group.

The results of the single environmental factor analysis for the different age groups are shown in Figure 3. For the middle-aged group (45–64), the former smokers (OR = 1.063, 95 CI%: 1.018, 1.110), and current smokers (OR = 1.063, 95 CI%: 1.018, 1.110) in the Low-NDVI group showed a significant risk of hypertension, and the association was significantly different in the different NDVI concentration groups (*P* < 0.05). In the stratified analysis of the PM_2.5_ concentration, the association between the former smokers and hypertension in the High-PM_2.5_ group was statistically significant, with an OR of 1.056 (95 CI%: 1.002, 1.113). However, there was no significant difference between this OR and the effect description of the Low-PM_2.5_ group (*P* = 0.760). The study further tested the difference between the significant effects observed in the low NDVI group in the middle age group and the elderly group. The results showed that the *P-*values for the difference of the former smokers and current smokers exposed to low green spaces in the different age groups were 0.881 and 0.299, respectively. Therefore, it was considered that there was no statistically significant difference in the effect description of the Low-NDVI groups among the different age groups. In addition, we did not observe any significant association between smoking and hypertension in the elderly group. Supplementary Table 1 shows the specific results of the stratified analysis of the single environmental factors for the different age groups.

### Dual environmental factor stratified results

Table 5 shows the results of the hierarchical analysis of the dual environmental factors. In order to facilitate the presentation of the results, the definition of each dual environmental factor subgroup is stated in Supplementary Table 2. In the Low-NDVI/High-PM_2.5_ subgroup, the current smokers and former smokers were significantly associated with hypertension, with an ORs of 1.064 (95 CI%: 1.012, 1.118) and 1.082 (95 CI%: 1.021, 1.146), respectively. No significant association between smoking and hypertension was observed in other subgroups. In order to test whether the significant effects observed in the Low-NDVI/High-PM_2.5_ group were significantly different from other subgroups, the study conducted a difference test for the OR of the Low-NDVI/High-PM_2.5_ group. For the current smokers, there was a significant difference in the risk of hypertension between the Low-NDVI/High-PM_2.5_ subgroup and the High-NDVI/Low-PM_2.5_ subgroup (*P* = 0.047). This means that there was a significant difference in the risk of hypertension between the former smokers who had been long-term exposed to low green spaces and high PM_2.5_ environments and those who had been long-term exposed to high green spaces and low PM_2.5_ particulate matter environments. For the former smokers of the Low-NDVI/High-PM_2.5_ subgroup, although the intensity of hypertension risk effect description was higher than that of the current smokers, the difference between this effect and the other subgroups was not significant (*P* > 0.05). The results of the model multicollinearity test showed that the GVIFs of the all the models were lower than two, which means that there was no multicollinearity problem in the dual environmental factors analysis.

**Table 5:** ORs of hypertension (with 95% confidence intervals, 95%CI) associated with smoking stratified by dual environmental factors^a^.

**Variable**	**Low-NDVI/Low-PM**_**2.5**_ **(***n** =*** **2,328)**			**Low-NDVI/High-PM**_**2.5**_ **(***n** =*** **2,930)**			**High-NDVI/Low-PM**_**2.5**_ **(***n** =*** **2,920)**			**High-NDVI/High-PM**_**2.5**_ **(***n** =*** **2,422)**		
	***n***	**OR (95%CI)**	**P for ORs**	***n***	**OR (95%CI)**	**P for ORs**	***n***	**OR (95%CI)**	**P for ORs**	***n***	**OR (95%CI)**	**P for ORs**
Never smoking	1,447	-		1,781	-		1,772	-		1,479	-	
Current smoker	612	1.046 (0.990,1.104)	0.659	786	1.064 (1.012,1.118)^**^	-	831	0.991 (0.944,1.041)	0.047	617	0.992 (0.939,1.047)	0.064
Former smoker	269	1.056 (0.992,1.124)^*^	0.581	363	1.082 (1.021,1.146)^***^	-	317	1.001 (0.948,1.057)	0.056	326	1.023 (0.963,1.088)	0.196

a Gender, age, education level, alcohol consumption, daily cigarette consumption, social activity, physical activity, sleep time, per capita GDP, NO_2_, and O_3_ were controlled as covariates in the model.

(1)

*** p < 0.01;

** p < 0.05;

* p < 0.10. (2) In all models, the GVIFs were < 1.860. (3) P for ORs < 0.05 means that there was a significant OR difference between this group and the Low-NDVI/High-PM_2.5_ group.

### Sensitivity analysis

The results of the two sensitivity analyses conducted with half a year and 2 years as the exposure periods are presented in Supplementary Tables 3, 4. The sensitivity analysis results were near to the results of the one-year exposure period, and some additional significant relationships were observed. In the stratified analysis of the single environmental factors that utilized 2 years as the exposure period, the risk of hypertension of the former smokers and current smokers in the Low-NDVI group was significant (OR = 1.075, 95 CI%: 1.030, 1.121), and their effect descriptions were both significantly different from that of the High-NDVI group. However, this conclusion was only applicable to the current smokers in the one-year exposure period. In addition, in the stratified analysis of the dual environmental factors with half a year or two as the exposure period, both the former smokers and current smokers in the Low-NDVI/High-PM_2.5_ group were significantly associated with hypertension, and their effect descriptions were significantly different from that of the High-NDVI/Low-PM_2.5_ group (P for ORs < 0.05).

## Discussion

To the best of our knowledge, this was the first study to explore the association between smoking and hypertension under different air pollution and green space exposures. Our findings showed that the effects of smoking on hypertension were diverse under different environmental exposure conditions. There was a significant relationship between smoking behavior and hypertension in the Low-NDVI group, and the effect value of this relationship was significantly different from that in the High-NDVI group. Furthermore, for the respondents exposed to low green spaces and high PM_2.5_ environments at the same time (Low-NDVI/High-PM_2.5_ group), their smoking behavior may lead to an increase in the risk of hypertension. Additionally, the description of this effect was significantly different from that of the High-NDVI/Low-PM_2.5_ group. In addition, for middle-aged people (45–64), the risk of hypertension caused by smoking was also more significant under long-term low green space environmental exposure. However, there was no significant difference in the effect among the different age groups.

For the last century, the relationship between smoking and hypertension has been repeatedly emphasized (35, 36), and the results of our study supported this broad consensus as well. However, we further found that under lower green space exposure and higher PM_2.5_ exposure, there was a more significant link between smoking and hypertension. Currently, there are few studies regarding the relationship between smoking and hypertension under different environmental exposure conditions. However, some studies that have investigated the relationship between environmental factors and hypertension have conducted stratified analysis on smoking that support our research results. A cross-sectional study found that the risk of hypertension of current smokers decreased significantly with an increase in the IQR (Inter quartile range) of green space exposure, and the degree of reduction was significantly different from that of people who had never smoked (*P* = 0.013) (37). A prospective cohort study found that with an increase in green space exposure, there was a difference in the effect of hypertension risk changes between smokers and non-smokers (38). Our study also observed a significant relationship between current smokers (OR = 1.058, 95 CI%: 1.020, 1.097) and hypertension in the low green space exposure group, and this effect was significantly different from the high green space exposure group (*P* = 0.011). Liu et al. found that every increase in the concentration of PM_2.5_ by one IQR (41.7 μg/m^3^) increased the risk of hypertension of smokers by 16% (95 CI%: 1.05,1.27), higher than that of never smokers (OR = 1.09,95 CI%: 1.02, 1.16) (21). For our study, Although the effect difference between the concentration levels was not statistically significant, the high PM2.5 group also showed a significant relationship between the former smokers and hypertension (OR = 1.052, 95 CI%: 1.008, 1.097). These similar results suggest that explaining the risk of hypertension from two dimensions of smoking behavior and environmental factors can control more confounding, thus providing a more accurate description of the effects. However, there are still few reports that comprehensively consider health behaviors and environmental factors, and more relevant studies need to be conducted to confirm the relationship between smoking and hypertension under different environmental exposure conditions.

The increased risk of hypertension caused by smoking behavior under adverse environmental exposure may be related to mental health. According to an investigation on some influencing factors of smoking prevalence, mental health and life stress caused by deterioration of the living environment may increase the prevalence of smoking behavior. Yang Chen et al. believed that alleviating life-related stress and insufficient satisfaction with the living environment were important motivations for urban residents to stop or reduce smoking (39). Timmermans et al. also mentioned that excessive smoking was a coping mechanism to reduce stress that was associated with living in an unpleasant environment (40). Interestingly, a study conducted in Canada found a 6% lower odds of poor self-rated mental health per increase in the interquartile range (0.12) of the NDVI (500 m buffer) (41), and another prospective cohort study also supported a negative correlation between green space exposure and work-related chronic stress (42). Additionally, a meta-analysis published in 2019 showed that the long-term exposure concentration of PM_2.5_ was significantly associated with depression (pooled OR = 1.102, 95% CI: 1.023, 1.189) (43), while the smoking cessation rate of depressed smokers was always considered to be lower than that of the general population (44). The results of these studies combined with the results of this study indicate that air pollution and green space exposure may induce residents' smoking behavior in the form of stress accumulation or anxiety. This would result in a difference in the hypertension risk among smokers under different environmental exposure conditions. More studies need to be conducted to demonstrate this conclusion.

We observed the most significant relationship between smoking and hypertension in the Low NDVI/High-PM_2.5_ group, and this may have been related to the pathogenesis of hypertension. Many studies have shown that the mechanism of reducing NDVI exposure and increasing PM_2.5_ exposure leading to increased risk of hypertension is similar to that of smoking leading to cardiovascular disease. Exposed in high green space for a long time may improve the immune regulation pathway, thus forming an antagonistic relationship with smoking behavior (45). Similar to the conclusion of our study, Jiang et al. also observed that the risk of hypertension of current smokers decreased significantly in higher green space exposure environments. They claim that long-term exposure to green spaces may expose residents to more microorganisms related to human immune regulation, thus activating the immune regulation system to reduce the risk of chronic inflammation (37), while and the mechanism of smoking affects systemic vascular resistance by inducing an inflammatory response, as has been reported by previous studies (46). In addition, long-term exposure to PM_2.5_ may promote vasoconstriction and weaken the ability of vasodilation (47), while nicotine leads to a rise in low-density lipoprotein and a decrease in high-density lipoprotein that also intensify vasoconstriction and accelerate the progression of blood epithelial cell injury (1). Oxidative stress induced by smoking may trigger cytokine release and systemic vascular inflammation caused by inflammatory cell adhesion and ultimately destroy the integrity of the endothelium as a protective barrier layer (48). Additionally, surface-bound reactive co-pollutants, such as transition metals and endotoxins, may enter the body through the pulmonary circulation with PM_2.5_ as a carrier, and this would also promote endothelial dysfunction in the form of oxidative stress, thus causing hypertension (49). In conclusion, green space exposure may antagonize the risk of hypertension caused by smoking through the immune pathway, while PM_2.5_ exposure and smoking have multiple synergistic effects on the pathogenesis of hypertension. The mechanism effect superposition of environmental factors and smoking behavior may be one reason why the Low NDVI/high PM_2.5_ group was observed to have the most significant effects, but this still needs to be verified by more relevant experiments.

In addition, our study observed in the age group analysis that middle-aged smokers aged 45–64 years had a higher risk of hypertension under low green space exposure. A survey on smoking influencing factors in 31 provinces in China showed that residents aged 40–59 were the primary group of Chinese smokers, and middle-aged people have a more serious tobacco dependence rate than the elderly (OR = 1.50, 95 CI%: 1.30, 1.70) (20). A 19-year follow-up investigation conducted in Asia showed that compared with the elderly, the attribution rate of CVD death caused by smoking was higher in men younger than 60 years old (50). However, middle-aged people were also reported as a high-risk group in the study of green space exposure and hypertension risk. A study conducted in Alpine valley showed that the diastolic blood pressure of participants aged 46–58 years decreased significantly under high green space exposure (value = −2.98, 95 CI%: −5.33, −0.63), while elderly participants (aged 58–81 years) had no significant relationship between blood pressure and green space exposure environment (51). However, some studies believed that the risk of smoking induced hypertension was not different among different age groups (9). However, a survey conducted in two European cities suggested that the risk of hypertension among people over 65 years old was more likely to decrease with an increase in green space exposure (19). The risk of smoking induced hypertension in the different age groups under the different environmental exposure conditions requires more research and exploration.

This study may have several strengths. First, this is the first study to explore the impact of smoking on the risk of hypertension under different environmental conditions. A description of the relationship between health behavior and the risk of chronic diseases under different environmental exposure conditions has rarely been reported in previous studies. Second, the study established a series of two factor subgroup analyses based on the median exposure concentrations of PM_2.5_ and the NDVI, and this may have reduced the potential confounding by or interaction with other environmental factors compared with the stratification of a single one (52). In addition, our study conducted a difference test for the effects of each environmental subgroup to ensure that the effect description difference of the different environmental conditions was reliable statistically. Third, this study was based on a large nationwide cohort in China (CHARLS), ensuring sufficient statistical power and generalizability of the results (21).

Several limitations of this study should be recognized. The PM_2.5_ exposure data were collected from fixed monitoring stations in cities. The summarizing of health data into a rough temporal and spatial range for the exposure analysis may have increased the uncertainty of environmental exposure and led to the wrong classification of the individual level exposure (53). Combining the address information of the interviewees and simulating the individual exposure assessment level through a land use regression model and other methods will make the description of the environmental variables of the interviewees more accurate (54). Second, as an important risk factor of CVD and hypertension, the BMI was not included in the model control in our study. Considering the association between obesity and CVD, the description of the relationship between smoking and hypertension in this study may have been affected. Third, the description of smoking behavior lacked detail. Due to the insufficient response rate or illogical data of the corresponding questionnaire items, the length of smoking was not included in our study for discussion. Although this study was sufficient to support the conclusion that smoking behavior has an effect on the increase in hypertension risk under different environmental conditions, there might still be a better classification scheme for the different types or degrees of smokers to explore the differences in effects. In addition, CHARLS did not record the frequency of respondents' green space use and the type of green space around the residence. To describe respondents' green space exposure more accurately, future relevant research should consider collecting respondents' subjective measurement information of green space, such as the self-reported quality of the neighborhood green space and the self-reported walking frequency in green spaces (55).

In conclusion, our research showed that smoking had different effects on hypertension under different environmental exposure conditions. Respondents exposed to low green spaces were more likely to suffer from hypertension due to smoking. Furthermore, the risk of hypertension caused by smoking in the Low NDVI/High PM_2.5_ group was significantly higher than that in the High NDVI/High PM_2.5_ group. In addition, for middle-aged people, the risk of hypertension caused by smoking was also more significant under long-term low green space environment exposure. Our results indicate that controlling environmental exposure can reduce the risk of smoking induced hypertension.

## Data availability statement

The raw data supporting the conclusions of this article will be made available by the authors, without undue reservation.

## Author contributions

LM provided the guidance of environmental epidemiology methods and statistical models for the study. QC completed the data analysis, chart drawing, and article writing. XM was responsible for the format and typesetting of the paper and participated in the analysis of tables and pictures. YG participated in the establishment of the database. JL provided guidance for the English writing of this article. All authors contributed to the article and approved the submitted version.

## Conflict of interest

The authors declare that the research was conducted in the absence of any commercial or financial relationships that could be construed as a potential conflict of interest.

## Publisher's note

All claims expressed in this article are solely those of the authors and do not necessarily represent those of their affiliated organizations, or those of the publisher, the editors and the reviewers. Any product that may be evaluated in this article, or claim that may be made by its manufacturer, is not guaranteed or endorsed by the publisher.
