# Supplementary material for: Association between smoking and hypertension under different PM2.5 and green space exposure: A nationwide cross-sectional study
Source: Front Public Health. 2022 Nov 17;10:1026648. doi: 10.3389/fpubh.2022.1026648 (PMC9712966; doi:10.3389/fpubh.2022.1026648)
Supplement: Supplementary file 1 [file Data_Sheet_1.pdf]

Supplementary Tables

Supplementary Table 1. ORs of hypertension (with 95% confidence intervals, 95%CI) associated with smoking stratified by a single environmental factor and age

Age	Variable	By NDVI [a]					By $PM_{2.5}$ [b]				
		Low-NDVI (n = 5258)		High-NDVI (n = 5342)		P for ORs	Low-$PM_{2.5}$ (n = 5248)		High-$PM_{2.5}$ (n = 5352)		P for ORs
		n	OR (95%CI)	n	OR (95%CI)		n	OR (95%CI)	n	OR (95%CI)	
Middle - aged (45 - 64)	Never smoking	2277	-	2314	-		2258	-	2333	-	
	Former smoker	1043	1.106 (1.049,1.167)***	1061	1.000 (0.949,1.052)	0.008	1076	1.044 (0.991,1.100)	1028	1.056 (1.002,1.113)**	0.760
	Current smoker	334	1.063 (1.018,1.110)***	358	0.961 (0.921,1.003)*	0.001	320	1.002 (0.960,1.046)	372	1.021 (0.978,1.066)	0.545
Elder (> 64)	Never smoking	951	-	937	-		961	-	927	-	
	Former smoker	355	1.055 (0.982,1.133)	387	1.052 (0.983,1.126)	0.955	367	1.030 (0.963,1.102)	375	1.088 (1.013,1.168)**	0.273
	Current smoker	298	1.056 (0.986,1.131)	285	1.052 (0.983,1.127)	0.939	266	1.064 (0.994,1.138)	317	1.057 (0.986,1.132)	0.894

[a] Gender, education level, alcohol consumption, daily cigarette consumption, social activity, physical activity, sleep time, per capita GDP, NO_2, $PM_{2.5}$, and O_3 were controlled as covariates in the model.

[b] Gender, education level, alcohol consumption, daily cigarette consumption, social activity, physical activity, sleep time, per capita GDP, NDVI, NO_2, and O_3 were controlled as covariates in the model.

Notes: (1) ***p < 0.01; **p < 0.05; *p < 0.10 (2) In all models, the GVIFs were less than 1.883. (3) P for ORs < 0.05 means that there was a significant difference in the ORs of the different exposure concentration groups.

Supplementary Table 2. Definition of each group in the dual environmental factor stratified analysis [a]

Group	NDVI exposure	$PM_{2.5}$ exposure
Low-NDVI/Low-$PM_{2.5}$	Below the median of the study population	Below the median of the study population
Low-NDVI/High-$PM_{2.5}$	Below the median of the study population	Higher than the median of the study population
High-NDVI/Low-$PM_{2.5}$	Higher than the median of the study population	Below the median of the study population
High-NDVI/High-$PM_{2.5}$	Higher than the median of the study population	Higher than the median of the study population

[a] The exposure period of the study population is one year

Supplementary Table 3. ORs of hypertension (with 95% confidence intervals, 95%CI) associated with smoking stratified by a single environmental factor in the different exposure periods

Period	Variable	By NDVI [a]					By $PM_{2.5}$ [b]				
		Low-NDVI		High-NDVI		P for ORs	Low-$PM_{2.5}$		High-$PM_{2.5}$		P for ORs
		n	OR (95%CI)	n	OR (95%CI)		n	OR (95%CI)	n	OR (95%CI)	
Half - year	Never smoking	3181	-	3298	-		3203	-	3276	-	
	Former smoker	635	1.070 (1.025,1.116)***	640	1.014 (0.974,1.055)	0.071	579	1.008 (0.967,1.051)	696	1.066 (1.023,1.111)***	0.061
	Current smoker	1388	1.056 (1.018,1.096)***	1458	0.993 (0.958,1.029)	0.018	1401	0.998 (0.962,1.035)	1445	1.047 (1.010,1.086)**	0.066
Two - year	Never smoking	3215	-	3264	-		3176	-	3303	-	
	Former smoker	643	1.075 (1.030,1.121)***	632	1.007 (0.967,1.049)	0.029	581	1.039 (0.997,1.083)*	694	1.039 (0.997,1.083)*	0.999
	Current smoker	1401	1.059 (1.021,1.098)***	1445	0.990 (0.955,1.026)	0.010	1419	1.031 (0.994,1.069)	1427	1.017 (0.981,1.055)	0.602

[a] Gender, education level, alcohol consumption, daily cigarette consumption, social activity, physical activity, sleep time, per capita GDP, NO_2, $PM_{2.5}$, and O_3 were controlled as covariates in the model.

[b] Gender, education level, alcohol consumption, daily cigarette consumption, social activity, physical activity, sleep time, per capita GDP, NDVI, NO_2, and O_3 were controlled as covariates in the model.

Notes: (1) ***p < 0.01; **p < 0.05; *p < 0.10 (2) In all models, the GVIFs were less than 1.806. (3) P for ORs < 0.05 means that there was a significant difference in the ORs of the different exposure concentration groups.

Supplementary Table 4. ORs of hypertension (with 95% confidence intervals, 95%CI) associated with smoking stratified by dual environmental factors in the different exposure periods [a]

Period	Variable	Low-NDVI/Low-$PM_{2.5}$			Low-NDVI/High-$PM_{2.5}$			High-NDVI/Low-$PM_{2.5}$			High-NDVI/High-$PM_{2.5}$		
		n	OR (95%CI)	P for ORs	n	OR (95%CI)	P for ORs	n	OR (95%CI)	P for ORs	n	OR (95%CI)	P for ORs
Half - year	Never smoking	1428	-		1753	-		1775	-		1523	-	
	Former smoker	265	1.025 (0.962,1.091)	0.074	370	1.108 (1.046,1.174)***	-	314	0.996 (0.942,1.052)	0.009	326	1.026 (0.967,1.089)	0.069
	Current smoker	593	1.024 (0.97,1.082)	0.138	795	1.083 (1.030,1.138)***	-	808	0.973 (0.927,1.022)	0.003	650	1.01 (0.958,1.065)	0.060
Two - year	Never smoking	1379	-		1836	-		1797	-		1467	-	
	Former smoker	281	1.059 (0.994,1.128)	0.549	362	1.087 (1.026,1.152)***	-	300	0.993 (0.935,1.054)	0.033	332	1.019 (0.964,1.077)	0.112
	Current smoker	609	1.042 (0.986,1.101)	0.497	792	1.069 (1.018,1.123)***	-	810	0.960 (0.910,1.014)	0.004	635	1.017 (0.968,1.067)	0.157

[a] Gender, age, education level, alcohol consumption, daily cigarette consumption, social activity, physical activity, sleep time, per capita GDP, NO_2 and O_3 were controlled as covariates in the model.

Notes: (1) ***p < 0.01; **p < 0.05; *p < 0.10. (2) In all models, the GVIFs were less than 1.822. (3) P for ORs < 0.05 means that there was a significant OR difference between this group and the Low-NDVI/High-$PM_{2.5}$ group.
